# Microtubule Dynamics Deregulation Induces Apoptosis in Human Urothelial Bladder Cancer Cells via a p53-Independent Pathway

**DOI:** 10.3390/cancers15143730

**Published:** 2023-07-22

**Authors:** Yiannis Drosos, Eumorphia G. Konstantakou, Aggeliki-Stefania Bassogianni, Konstantinos-Stylianos Nikolakopoulos, Dimitra G. Koumoundourou, Sophia P. Markaki, Ourania E. Tsitsilonis, Gerassimos E. Voutsinas, Dimitrios Valakos, Ema Anastasiadou, Dimitris Thanos, Athanassios D. Velentzas, Dimitrios J. Stravopodis

**Affiliations:** 1 Section of Cell Biology and Biophysics, Department of Biology, School of Science, National and Kapodistrian University of Athens (NKUA), 15701 Athens, Greece; sbasogianni@gmail.com (A.-S.B.); ksnikolakop@biol.uoa.gr (K.-S.N.); dimikoum@biol.uoa.gr (D.G.K.); smarkak@biol.uoa.gr (S.P.M.); tveletz@biol.uoa.gr (A.D.V.); 2 Massachusetts General Hospital Cancer Center (MGHCC), Harvard Medical School, Boston, MA 02114, USA; ekonstantakou@mgh.harvard.edu; 3 Section of Animal and Human Physiology, Department of Biology, School of Science, National and Kapodistrian University of Athens (NKUA), 15701 Athens, Greece; rtsitsil@biol.uoa.gr; 4 Laboratory of Molecular Carcinogenesis and Rare Disease Genetics, Institute of Biosciences and Applications (IBA), National Center for Scientific Research (NCSR) “Demokritos”, 15310 Athens, Greece; mvoutsin@bio.demokritos.gr; 5 Center of Basic Research, Biomedical Research Foundation of the Academy of Athens (BRFAA), 11527 Athens, Greece; dvalakos@bioacademy.gr (D.V.); anastasiadou@bioacademy.gr (E.A.); thanos@bioacademy.gr (D.T.)

**Keywords:** apoptosis, bladder, cancer, microtubule, p53, paclitaxel, urothelium

## Abstract

**Simple Summary:**

Bladder cancer (BLCA) is considered as a highly prevalent disease that is strongly associated with elevated morbidity, mortality, and cost. Strategies designed to be targeting critical components and processes orchestrating BLCA cells’ evolutionary trajectories, towards metastasis and chemoresistance, are necessitated to be promptly developed, and successfully pass the proof-of-principle tests in pre-clinical models and clinical trials. To this direction, DepMap and PRISM project-derived findings, combined with transcriptomics and epigenetic data analysis, have herein unveiled the cardinal roles of microtubule dynamics in the survival and growth of BLCA cells. Most importantly, they can foresee the therapeutic promise of pathway’s targeted perturbation, with paclitaxel single scheme and paclitaxel-containing drug-cocktails opening new therapeutic windows for the disease.

**Abstract:**

Bladder cancer (BLCA) is the sixth most common type of cancer and has a dismal prognosis if diagnosed late. To identify treatment options for BLCA, we systematically evaluated data from the Broad Institute DepMap project. We found that urothelial BLCA cell lines are among the most sensitive to microtubule assembly inhibition by paclitaxel treatment. Strikingly, we revealed that the top dependencies in BLCA cell lines include genes encoding proteins involved in microtubule assembly. This highlights the importance of microtubule network dynamics as a major vulnerability in human BLCA. In cancers such as ovarian and breast, where paclitaxel is the gold standard of care, resistance to paclitaxel treatment has been linked to p53-inactivating mutations. To study the response of BLCA to microtubule assembly inhibition and its mechanistic link with the mutational status of the p53 protein, we treated a collection of BLCA cell lines with a dose range of paclitaxel and performed a detailed characterization of the response. We discovered that BLCA cell lines are significantly sensitive to low concentrations of paclitaxel, independently of their p53 status. Paclitaxel induced a G2/M cell cycle arrest and growth inhibition, followed by robust activation of apoptosis. Most importantly, we revealed that paclitaxel triggered a robust DNA-damage response and apoptosis program without activating the p53 pathway. Integration of transcriptomics, epigenetic, and dependency data demonstrated that the response of BLCA to paclitaxel is independent of p53 mutational signatures but strongly depends on the expression of DNA repair genes. Our work highlights urothelial BLCA as an exceptional candidate for paclitaxel treatment. It paves the way for the rational use of a combination of paclitaxel and DNA repair inhibitors as an effective, novel therapeutic strategy.

## 1. Introduction

Bladder cancer (BLCA) is a devastating disease affecting over 500,000 individuals worldwide and is responsible for over 200,000 deaths on an annual basis [1,2]. BLCA is classified as either a low-grade, non-muscle-invasive disease or a high-grade, muscle-invasive disease with a higher chance of metastasis to distal organs [2,3]. Several driver mutations have been identified in low-grade papillary tumor development, such as in the *FGFR3*, *H-Ras*, and *mTOR* pathway member genes. In contrast, the progression to high-grade invasive urothelial carcinoma is correlated with loss of function of *p53* and *Rb* tumor-suppressor network genes [4]. Moreover, an integrated study of 131 invasive bladder carcinomas revealed dysregulation of PI3K/Akt/mTOR and RTK/Ras/MAPK pathways in more than 40% of all tumors analyzed [5].

Current treatment regimens, such as chemotherapy and radiotherapy, surgery, and, more recently, immunotherapy, have improved survival but, in many cases, are associated with severe side effects or the development of secondary resistance to therapy [6,7]. Moreover, developing new targeted therapies is a very tedious and uncertain process, and it can take more than 10 years for a new agent to reach the clinic. Therefore, there is a great need to develop better-targeted treatments for BLCA, ideally by utilizing or repurposing FDA-approved compounds.

In the effort to identify novel therapeutic vulnerabilities in cancer and utilize/repurpose existing drugs, the Cancer Dependency Map (DepMap) and the Profiling Relative Inhibition Simultaneously in Mixtures (PRISM) projects have made tremendous progress [8,9,10]. DepMap utilizes the power of high-throughput genome-wide CRISPR screens across hundreds of cancer cell lines and across a wide spectrum of malignancy types to identify genetic vulnerabilities unique to each cancer type [9]. In contrast, the PRISM project exploits high-throughput chemical screens to repurpose and/or identify novel therapeutic compounds [10].

By investigating the data generated by the DepMap and PRISM projects and combining it with epigenetic and transcriptomics data analysis, we uncovered the importance of microtubule dynamics for the survival of BLCA cells and the therapeutic potential of perturbing this pathway.

## 2. Materials and Methods

### 2.1. Cell Lines and Culture Conditions

The four human cell lines of the present study, RT4, RT112, T24, and TCCSUP, were derived from urothelial carcinomas of the bladder. RT4 cells were purchased from ECACC-Sigma-Aldrich (St. Louis, MO, USA). RT112 cells were kindly and generously provided by Professor J.R. Masters (London, UK). T24 and TCCSUP cell lines were obtained from ATCC-LGC Standards GmbH (Wesel, Germany). The growth and manipulation of RT4, RT112, T24, and TCCSUP human urothelial BLCA cell lines used in this study have been previously described [11]. In brief, cell cultures were grown in complete DMEM medium, supplemented with 10% FBS (Thermo Fisher Scientific Inc.—Life Technologies—Gibco, Waltham, MA, USA), at 37 °C and 5% CO_2_, and subcultured every 2–3 days.

### 2.2. Cell Viability MTT Assays

Cells were seeded onto 48-well plates at a confluency of ~90% and treated with different paclitaxel doses for 24 and 48 h. Then, cells were incubated with MTT solutions for 4 h. The formazan crystals produced were dissolved in pure isopropanol. Spectrophotometric absorbance was measured in a Dynatech MR5000 ELISA microtiter plate reader (Dynatech Laboratories, Chantilly, VA, USA) at 550 nm, with 630 nm as the reference wavelength. Each assay was repeated three times, using three wells per experimental condition.

### 2.3. Flow Cytometry for Cell Cycle Analysis

Control and paclitaxel-treated cells were incubated with 10 μL of 7AAD solution for 15 min at +4 °C in the dark. All cell preparations were analyzed within 30 min by flow cytometry using a Beckman Coulter Cytomics FC500 cell sorter (Beckman Coulter Inc., Brea, CA, USA).

### 2.4. Western Blotting

Immunoblot experiments were performed as previously described [11]. In brief, ~40 μg of whole-cell protein extracts were separated by SDS-PAGE in 10–15% gels and subsequently transferred onto nitrocellulose membranes (Whatman Schleicher & Schuell GmbH, Dassel, Germany). The blocking process was carried out by treating membranes with TBST containing 5% NFM (or BSA) for 2 h at room temperature. Each primary antibody was added at a concentration of 1:1000 for 1 h at room temperature and 16 h at +4 °C. In contrast, at room temperature, the appropriate IgG-HRP secondary antibody (anti-rabbit or anti-mouse) was used at a dilution of 1:2000 for 2 h. Immunoreactive bands were visualized by ECL reactions, following the manufacturer’s instructions. Actin was used as a protein of reference.

### 2.5. RT-PCR

Total RNA from control and paclitaxel-treated cells was extracted following a Trizol-based protocol (Thermo Fisher Scientific Inc.—Life Technologies—Ambion, Waltham, MA, USA). RNA was reverse transcribed using an oligo-[dT]_12–18_ primer and the MMLV enzyme (Thermo Fisher Scientific Inc.—Life Technologies—Invitrogen, Waltham, MA, USA). cDNA was amplified by sqPCR with a Biometra T3000 Thermocycler (Biometra GmbH, Goettingen, Germany) using gene-specific oligonucleotide primers (Table S1). According to standard procedures, PCR fragments were resolved into 2–3% agarose gels. *GAPDH* served as a gene of reference.

### 2.6. DepMap Data Analysis

Genome-scale, CRISPR-Cas9-mediated screening has been previously described [12,13]. The most recent Avana CRISPR release (DepMap public 23Q2+Score, Chronos), which includes 32 BLCA cell lines and over 1000 lines of other lineages, screened with the Avana library, was used (data available at the DepMap Portal: https://depmap.org/portal, accessed on 23 March 2023). Gene effect difference was calculated as a T-statistic from the DepMap portal, as described in [13]. The primary PRISM repurposing dataset (https://depmap.org/repurposing, accessed on 27 March 2023) includes data from 4518 compounds screened against 578 cell lines [10]. In contrast, the secondary screen includes data from 1448 compounds screened against 499 cell lines in an 8-step, 4-fold (4×) dilution scheme, with 10 μM being the highest concentration tested [14]. The cell lines used in the PRISM screens are a subfraction of the total cell lines used in the CRISPR screen. Specifically for BLCA, only 24 out of the 32 BLCA cell lines in DepMap were successfully barcoded and included in the PRISM screen [10].

### 2.7. ATAC-Seq. of Primary Human Tumors

Normalized bigWig files containing ATAC-seq. reads from bladder, breast, lung, uterine, cervical, and kidney cancers were downloaded from the NCI portal, as described in [15]. Normalized ATAC-seq. reads were visualized using the IGV browser [16].

### 2.8. Statistical Analysis

Statistical analyses for DepMap and PRISM data were generated and obtained from the DepMap portal. Using GraphPad Prism v.9, the statistical significance of differences in sqPCR was determined using a one-way ANOVA with Tukey’s test for multiple hypothesis correction. A *p*-value < 0.05 was considered statistically significant.

## 3. Results

### 3.1. Urothelial Bladder Cancer Cells Are Significantly Sensitive to Microtubule Assembly Inhibition

**Figure 1:**
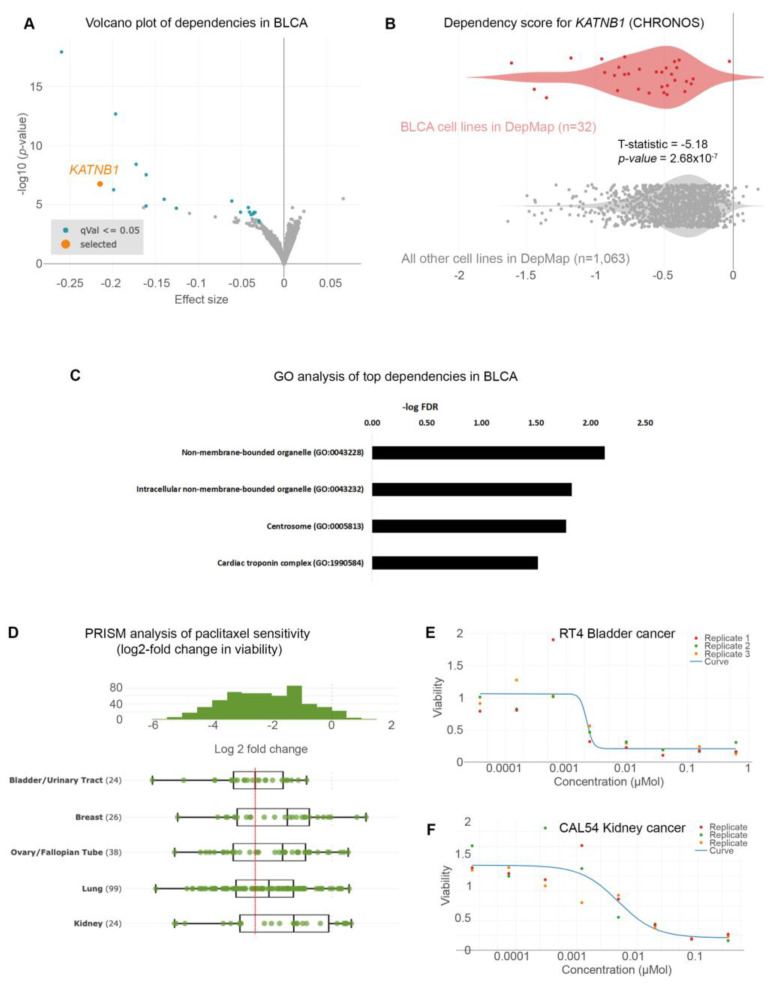
Urothelial bladder cancer (UBLCA) cells are extremely sensitive to microtubule assembly inhibition. (**A**) Genome-scale CRISPR-Cas9 screens of 32 BLCA cell lines. Each circle represents a gene. The *x*-axis represents the mean difference of the CHRONOS dependency scores in BLCA cell lines compared to all the other ones. Negative dependency scores indicate that BLCA cells require that gene, whereas positive scores suggest that the gene suppresses BLCA growth. Blue-colored circles represent significant negative dependencies, and of the top dependencies, *KATNB1* is colored orange. Statistical significance was calculated as −log10 (*p*-value) from two-sided *t*-tests with Benjamini–Hochberg correction. (**B**) Two class comparisons for *KATNB1*; one of the most significant dependencies highlighted in (**A**) between the 32 BLCA cell lines compared to all other cell lines in DepMap (n = 1063). (**C**) Gene ontology analysis of the 49 genes enriched as statistically significant dependencies in BLCA cells in (**A**). Only pathways with FDR < 0.05 are shown. (**D**) PRISM drug sensitivity results for paclitaxel are depicted as log2-fold changes in abundance of barcoded cells at d5 vs. d0. The results for bladder (urinary tract) cancer cells are shown compared to breast, ovarian (fallopian tube), lung, and kidney cancers. Breast, ovarian, and lung cancers are among the most sensitive, and paclitaxel is currently used as a standard of care. In contrast, kidney cancer cells are among the least sensitive and are depicted as controls. (**E**,**F**) Representative dose–response curves for paclitaxel in BLCA (RT4) and kidney cancer (CAL54) cell lines. T-statistic and *p*-value were obtained from the DepMap portal.

Given the poor prognoses of patients suffering from BLCA, we sought to identify gene dependencies and small molecules to which BLCA cells are preferentially sensitive by interrogating the DepMap project databases [10,13,14,17,18,19,20,21]. We first examined the gene dependencies across 1095 cell lines. We focused on dependencies specific to BLCA cell lines (n = 32) compared to all other cell lines in the DepMap database (n = 1063). We identified 35 statistically significant gene dependencies (Figure 1A and Table S2). The DepMap project utilizes genome-wide CRISPR screens across hundreds of cancer cell lines spanning all disease types. Each cell line is stably infected with a library of small-guide RNA (sgRNA) targeting all known human genes, plus cutting and non-cutting controls. The cells are stably infected with the lentiviral library and cultured for 2 weeks, and the abundance of each sgRNA is calculated at the end of the experiment compared to the beginning (day 0: d0). The effect of each sgRNA is calculated with an algorithm considering the cutting efficiency of each sgRNA and CRISPR-cutting-induced toxicity and is presented as the CHRONOS score [20,21]. A negative log2-fold change in the CHRONOS score for a certain gene means that, at the end of the experiment, the abundance of this sgRNA was reduced compared to d0. This means that deleting that gene is toxic. The CHRONOS score is calculated for all genes in each cell line. When focusing on BLCA cell lines, the top dependencies included genes encoding proteins involved in microtubule dynamics, such as KATNB1 (Katanin Regulatory Subunit B1), which participates in a complex that severs microtubules in an ATP-dependent fashion (Figure 1A,B). This suggests that this pathway is a specific vulnerability in BLCA cells. Ontology analysis revealed that the genes emerging as dependencies in BLCA are enriched in pathways related to centrosomes (Figure 1C and Table S3). This highlights the importance of microtubule assembly and dynamics for the survival of BLCA cells.

We analyzed the results from the PRISM repurposing drug screens to further identify therapeutic agents that could selectively target BLCA cells [10,14]. We focused our analysis on the primary PRISM screen, where the results are plotted as log2-fold changes in abundance of barcoded cells at day 5 (d5) compared to day 0 (d0) [14]. We first compared the sensitivity of BLCA cells to paclitaxel against cancers where paclitaxel is a standard of care, such as breast, ovarian, and lung carcinomas. We found that BLCA cells (n = 24) were among the most sensitive across all the cell lines (Figure S1) and more sensitive than breast, ovarian, and lung cancers (Figure 1D). Kidney cancer cells were among the least sensitive cell lines to paclitaxel (Figure 1D and Figure S1) and were used as a negative control hereafter. Representative dose–response curves from a BLCA and a kidney cancer cell line showed that BLCA cells are sensitive even to very low concentrations of paclitaxel. In contrast, kidney cancer cells can tolerate higher drug doses (Figure 1E,F).

**Figure 2:**
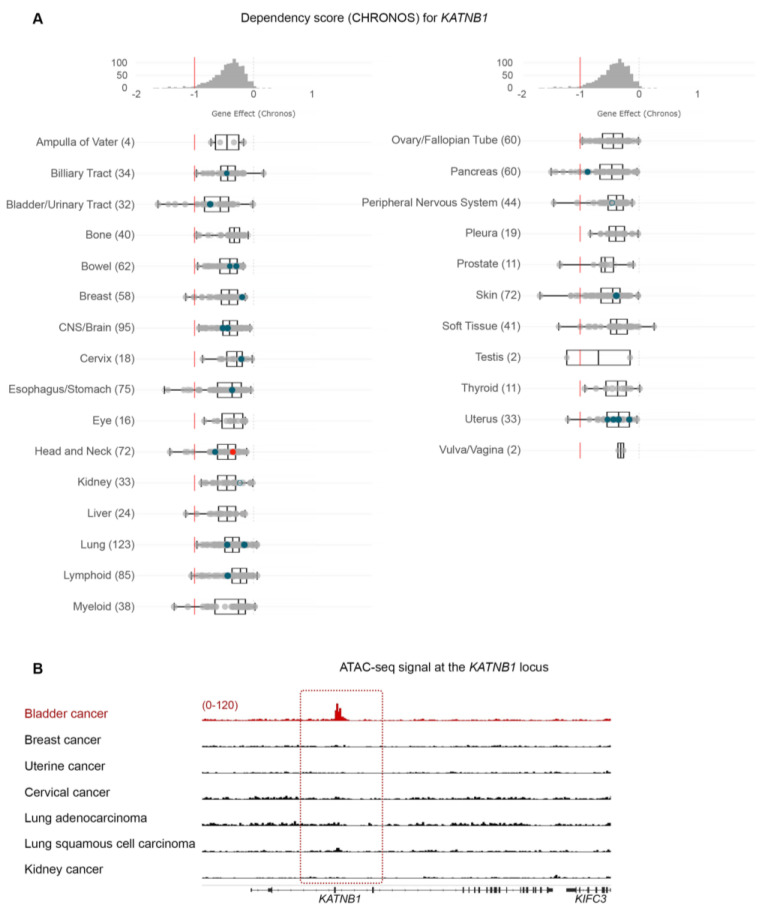
Microtubule-associated proteins are revealed as vulnerabilities in BLCA cells. (**A**) CHRONOS score for the *KATNB1* gene across all cancer cell lines and types in DepMap. BLCA cells are among the most dependent cancer types. (**B**) Normalized ATAC-seq. tracks of the *KATNB1* gene locus in 7 representative samples from various cancer types predicted to depend on *KATNB1*. Kidney cancer is used as a negative control.

Given the high sensitivity of BLCA cells to paclitaxel, especially compared to cancers that are considered sensitive to the drug (breast, ovarian, and lung), we further investigated the dependency of BLCA cells on microtubule assembly-associated proteins compared to all other cell lines (Figure 1B and Figure 2). Using *KATNB1* as a readout, the DepMap data suggest that BLCA cells are presented as the most sensitive to *KATNB1* perturbation (sgRNA targeting) compared to all other cell lines analyzed (Figure 2A).

Cancer vulnerabilities often underlie dependency on a gene due to its amplification or transcriptional abundance [22,23]. Therefore, we hypothesized that the dependence of BLCA cells on *KATNB1* will rely on a specific chromatin state that will facilitate abundant transcription of *KATNB1* compared to other cancer cells. We systematically investigated the open chromatin at the *KATNB1* locus to test our hypothesis using normalized ATAC-seq. data from a large collection of primary human cancers [15]. Aside from BLCA, we focused on breast, uterine, cervical, and lung cancers. We also investigated kidney cancer as a negative control, similar to our paclitaxel sensitivity analysis (Figure 2B). Our analysis demonstrates that BLCA cells have selectively open chromatin at the *KATNB1* gene locus compared to the other cases examined (Figure 2B).

Collectively, our analysis suggests that BLCA cells are preferentially sensitive to genetic and/or pharmacological perturbations of microtubule dynamics.

### 3.2. Paclitaxel Treatment Induces Cell Cycle Arrest and Growth Inhibition in Bladder Cancer Cells

Our analysis of the DepMap data and chromatin accessibility suggests that BLCA cells are sensitive to microtubule dynamics perturbations. To further confirm our findings, we used a collection of BLCA cell lines derived from different disease stages and treated them with a dose range of paclitaxel for 24 and 48 h. RT4 cell line originates from a grade I carcinoma, RT112 from grade II, T24 from grade III, and TCCSUP from grade IV. RT4 and RT112 have wild-type *TP53*, whereas T24 and TCCSUP carry *TP53* inactivating mutations and lack p53 functional protein form(s).

**Figure 3:**
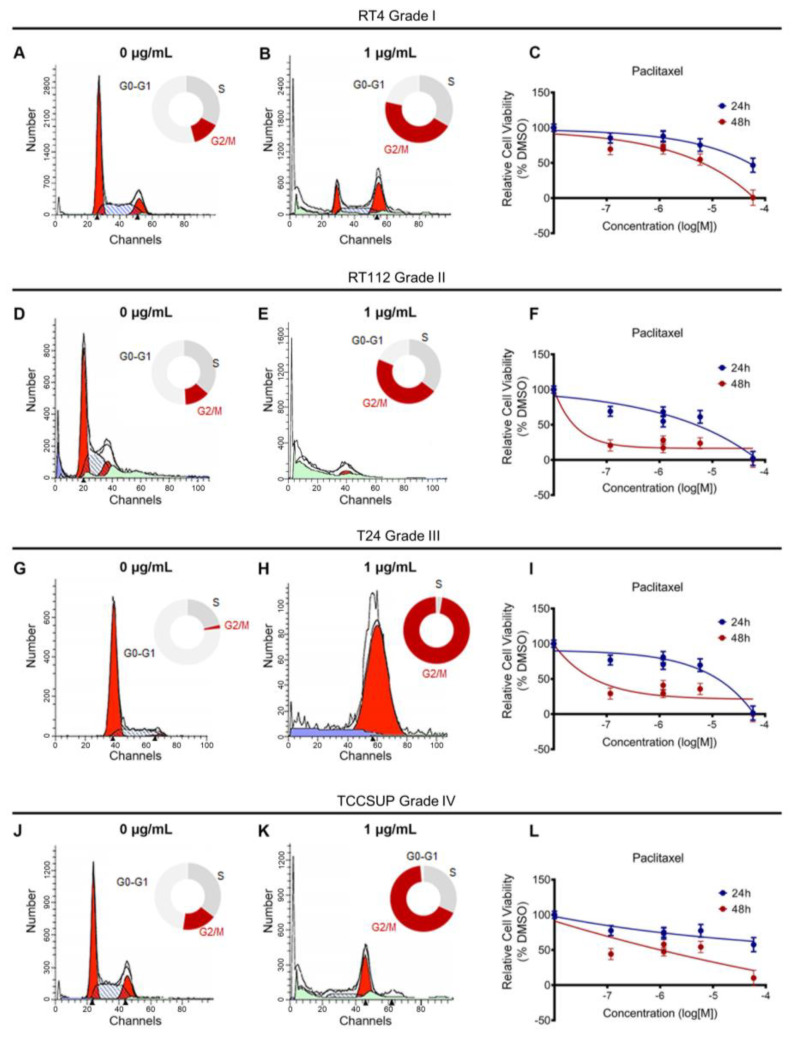
Paclitaxel treatment induces cell cycle arrest and growth inhibition in BLCA cells. (**A**,**B**) Flow cytometry analysis of RT4 cells (malignancy grade I) either untreated (**A**) or treated with 1 μg/mL paclitaxel (**B**). (**C**) Sensitivity of RT4 cells to paclitaxel after 24 and 48 h of treatment, as assessed by an MTT assay. (**D**,**E**) Flow cytometry analysis of RT112 cells (malignancy grade II) either untreated (**D**) or treated with 1 μg/mL paclitaxel (**E**). (**F**) Sensitivity of RT112 cells to paclitaxel after 24 and 48 h of treatment, as assessed by an MTT assay. (**G**,**H**) Flow cytometry analysis of T24 cells (malignancy grade III) either untreated (**G**) or treated with 1 μg/mL paclitaxel (**H**). (**I**) Sensitivity of T24 cells to paclitaxel after 24 and 48 h of treatment, as assessed by an MTT assay. (**J**,**K**) Flow cytometry analysis of TCCSUP cells (malignancy grade IV) either untreated (**J**) or treated with 1 μg/mL paclitaxel (**K**). (**L**) Sensitivity of TCCSUP cells to paclitaxel after 24 and 48 h of treatment, as assessed by an MTT assay. Flow cytometry experiments were performed in triplicate, and representative graphs were shown. MTT assays were performed in three biological replicates, with the graphs depicting the median values and error bars representing the standard error of each biological replicate.

Paclitaxel inhibits microtubule depolymerization, which is critical for mitotic spindle assembly. To test the effects of paclitaxel on the cell cycle, we treated RT4, RT112, T24, and TCCSUP cell lines with a dose range of the drug for 24 h. We also analyzed the effects using Fluorescent Activated Cell Sorting (FACS)-based cell cycle analysis. We found that a low concentration of paclitaxel-induced a robust cell cycle arrest at the G2/M phase in all cell lines analyzed, independently of their malignancy grade or p53 status (Figure 3A,B,D,E,G,H,J,K and Figure S2).

To further investigate the effects of paclitaxel on survival and growth, we treated our panel of BLCA cell lines with a dose range of the drug for either 24 or 48 h and monitored their survival and growth using an MTT assay. Consistent with the effects of paclitaxel on the cell cycle, drug treatment also induced growth inhibition. Although the results varied among the four cell lines tested, all cells exhibited significant growth inhibition 48 h after treatment, especially with the higher drug concentrations (Figure 3C,F,I,L).

Therefore, our in vitro study is in accordance with the in silico analysis regarding BLCA cells’ sensitivity to microtubule dynamics inhibition.

### 3.3. Paclitaxel Treatment Induces Apoptosis in Bladder Cancer Cells

**Figure 4:**
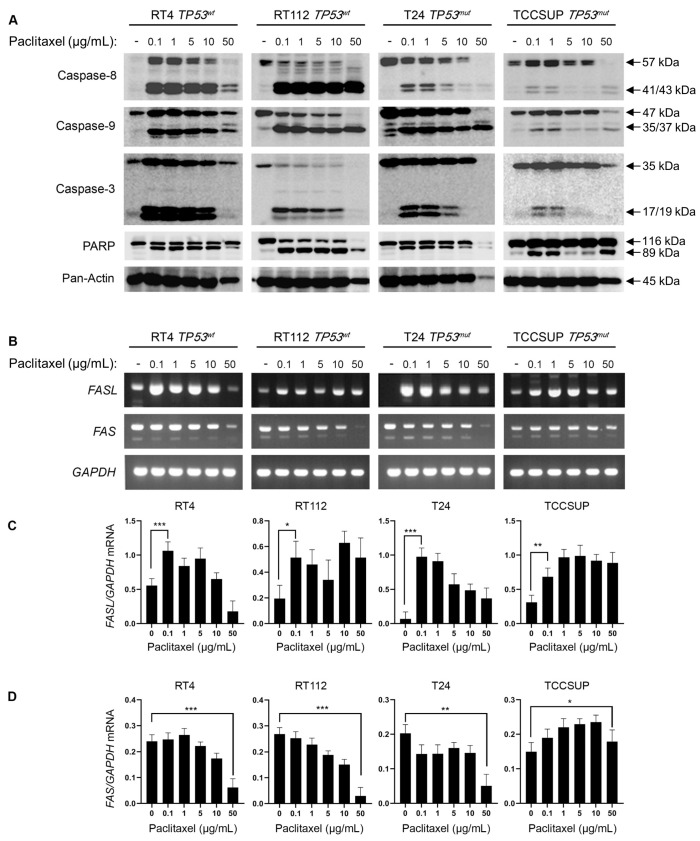
Paclitaxel treatment induces apoptosis in BLCA cells. (**A**) Immunoblot analysis of whole-cell protein extracts obtained from RT4, RT112, T24, and TCCSUP cells, seeded at ~60% confluency and exposed to the indicated doses (0, 0.1, 1, 5, 10, and 50 μg/mL) of paclitaxel for 24 h. Protein extracts were blotted for Caspase-8, -9, and -3 and PARP to assess apoptosis and for Pan-Actin as a reference protein (n = 3 biological replicates). (**B**) RT-sqPCR analysis of total RNA extracted from RT4, RT112, T24, and TCCSUP cells, seeded at ~60% confluency and exposed to the indicated doses (0, 0.1, 1, 5, 10, and 50 μg/mL) of paclitaxel for 24 h. Expression of *FASL* and *FAS* mRNA was analyzed to assess the induction of apoptosis. *GAPDH* mRNA expression was used as a control (n = 3 biological replicates). (**C**,**D**) Densitometry analysis of the obtained RT-sqPCR results (**B**). The density of *FASL* and *FAS* RT-sqPCR bands was analyzed and normalized to the density of *GAPDH* mRNA for each cell line and condition tested. Immunoblot and RT-sqPCR experiments were performed in triplicate, and representative images were shown. A one-way ANOVA with Tukey’s test for multiple hypothesis correction ensures the statistical significance of the results. Error bars represent means ± SD (n = 3 biological replicates). * *p* < 0.05, ** *p* < 0.01, and *** *p* < 0.001. See Supplementary Material for the original image of the Western Blots.

To investigate the induction of caspase-mediated apoptosis in BLCA cells following exposure to paclitaxel, we treated the panel of RT4, RT112, T24, and TCCSUP cell lines with varying doses of the drug for 24 h. We analyzed the cleavage profiles of Caspase-8, -9, -3, and PARP proteins using Western immunoblotting analysis. Our results clearly demonstrate that paclitaxel treatment induces cleavage of the caspase repertoire and the typical PARP substrate (Figure 4A). Although the level of caspase activation varied across the cell lines used, the cleaved forms of Caspase-8, -9, -3, and PAPR could be readily detected in all the cell lines tested. This strongly suggests that paclitaxel induces apoptosis in BLCA cells independently of their malignancy grade (I–IV) or p53 functional status (wild-type or mutated/inhibited). Interestingly, the caspase cleavage/activation induction level proved to correlate with the histological grade of the tumor each cell line derived from. A greater level of apoptosis activation was detected in the grade I RT4 cells, whereas a lower activation level was observed in the high-grade IV TCCSUP cells (Figure 4A).

To further test the induction of apoptosis in BLCA cells in response to paclitaxel, we used semi-quantitative (sq) RT-PCR as an orthogonal assay. We analyzed the expression of several genes encoding proteins involved in apoptotic cell death (Figure 4B–D and Figure S3). RT-sqPCR analysis showed that all the cell lines tested express high levels of apoptosis-related genes, such as *FASL*, *FAS*, *CIAP-1*, *CIAP-2*, *XIAP*, and *SURVIVIN* (Figure 4B–D and Figure S3). Most importantly, paclitaxel treatment induced a significant upregulation of *FASL* mRNA synthesis/accumulation in all BLCA cell lines tested, even at lower drug concentrations (Figure 4B–D). Therefore, it underpins the activation of a FASL-dependent apoptotic program.

Overall, our immunoblot and RT-sqPCR results so far demonstrate that paclitaxel treatment induces a robust apoptotic program in all BLCA cell lines tested, further confirming the sensitivity of BLCA cells to microtubule dynamics perturbation.

### 3.4. Bladder Cancer Cells Have Selectively Open Chromatin at the BCL2 Locus and Undergo BLC2 Downregulation upon Paclitaxel Treatment

**Figure 5:**
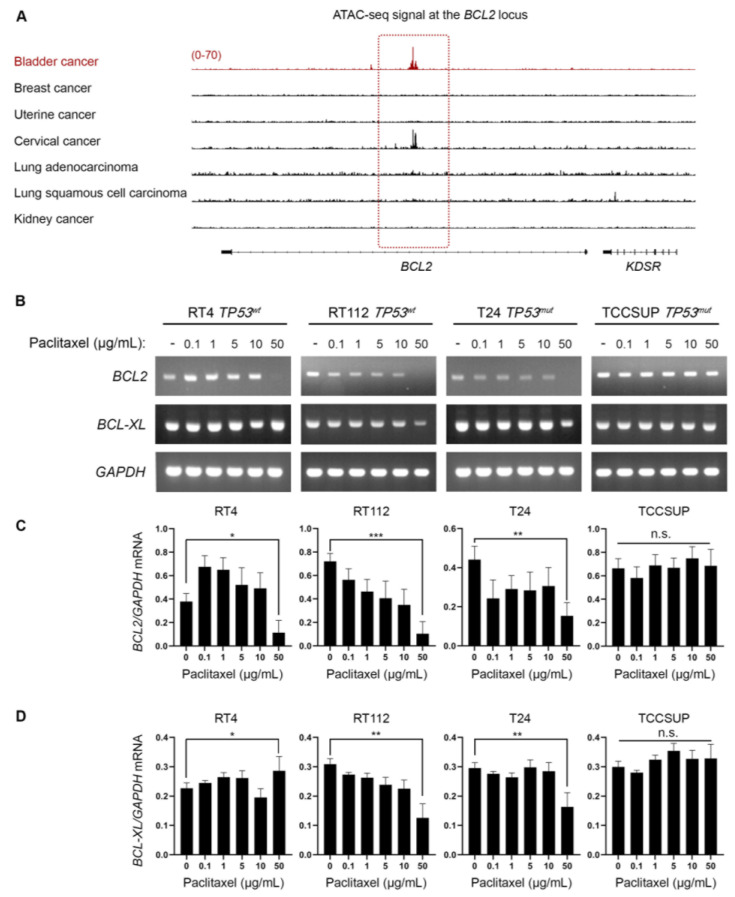
BLCA cells have a selectively open chromatin state at the *BCL2* locus and undergo *BLC2* downregulation upon paclitaxel treatment. (**A**) Normalized ATAC-seq. tracks of the *BCL2* locus in 7 representative samples from various cancer types predicted to be dependent on *BCL2* and sensitive to paclitaxel. Kidney cancer is used as a negative control. (**B**) RT-sqPCR analysis of total RNA extracted from RT4, RT112, T24, and TCCSUP cells, seeded at ~60% confluency and exposed to the indicated doses (0, 0.1, 1, 5, 10, and 50 μg/mL) of paclitaxel for 24 h. Expression of *BCL2* and *BCL-XL* mRNA was analyzed to assess the induction of apoptosis. *GAPDH* mRNA expression was used as a control (n = 3 biological replicates). (**C**,**D**) Densitometry analysis of the obtained RT-sqPCR results (**B**). The density of *BCL2* and *BCL-XL* RT-sqPCR bands was analyzed and normalized to the density of *GAPDH* mRNA for each cell line and condition examined. A one-way ANOVA with Tukey’s test for multiple hypothesis correction ensures the statistical significance of the results. Error bars represent means ± SD (n = 3 biological replicates) (n.s.: non-significant). * *p* < 0.05, ** *p* < 0.01, and *** *p* < 0.001. See Supplementary Material for the original images of the Western Blots.

Besides the well-characterized activity of paclitaxel to bind to microtubules and inhibit their depolymerization, it has been demonstrated to directly bind to BCL2 and inhibit its anti-apoptotic activity [24,25,26,27]. Given that our in vitro and in silico/DepMap-based analysis revealed that BLCA cell lines are extremely sensitive to paclitaxel, we sought to investigate whether the mechanism of action involves perturbation of BCL2 expression. In cancers addicted to BCL2, super-enhancers are formed that can maintain an open chromatin state at the *BCL2* locus and sustain its expression [28]. Then, we asked if there was preferentially open chromatin at the *BCL2* locus in BLCA cells that could suggest an oncogenic addiction to BCL2 expression. To answer our question, we investigated the open chromatin status of BLCA compared to breast, uterine, cervical, lung, and kidney cancers. We found that BLCA cells have preferentially open chromatin at the *BCL2* locus by plotting the normalized ATAC-seq. reads for each type of cancer (Figure 5A). Besides BLCA, cervical cancer cells also exhibited a strong ATAC-seq. signal at the *BCL2* locus, but neither breast (and uterine) nor lung cancer demonstrated such a signal, with kidney cancer serving as a negative control (Figure 5A).

To further test whether paclitaxel treatment perturbs BLC2, we analyzed the synthesis/accumulation of *BCL2* mRNA using RT-sqPCR in our panel of BLCA cells treated with paclitaxel for 24 h. Our results revealed that paclitaxel treatment downregulated the expression of the *BCL2* gene at the highest concentration tested, with RT4, RT112, and T24 cells presenting the most remarkable effect (Figure 5B–D).

Taken together, our results strongly suggest that BLCA cells depend on BCL2 gene activity due to a selective open chromatin status, and paclitaxel treatment results in downregulating *BCL2* mRNA levels.

### 3.5. Paclitaxel Treatment Induces DNA Damage without p53 Activation

**Figure 6:**
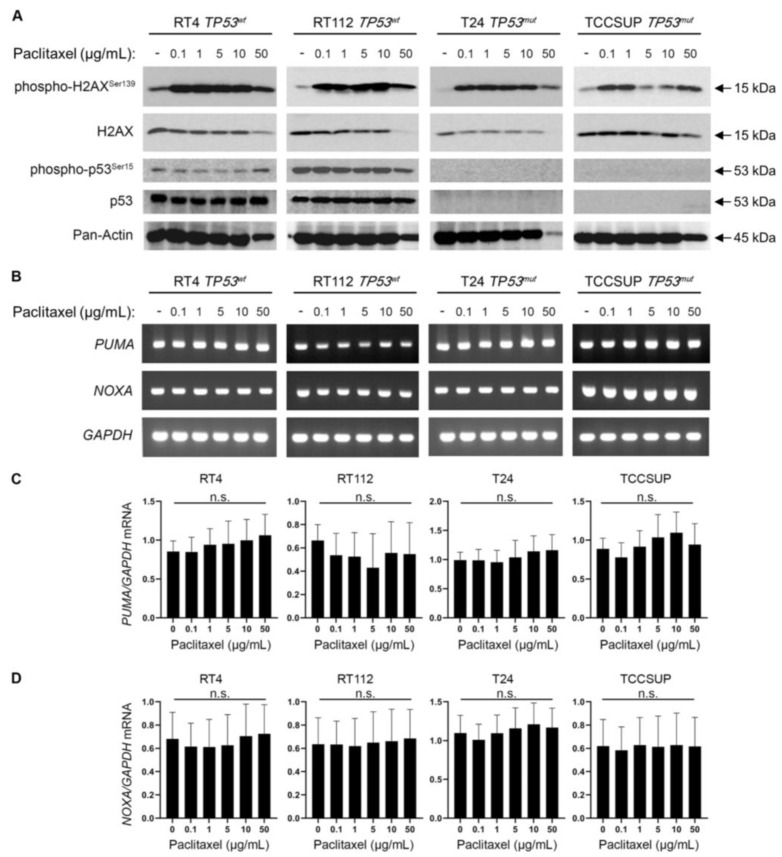
Paclitaxel treatment induces DNA damage without activating p53. (**A**) Immunoblot analysis of whole-cell protein extracts obtained from RT4, RT112, T24, and TCCSUP cells, seeded at ~60% confluency and exposed to the indicated doses (0, 0.1, 1, 5, 10, and 50 μg/mL) of paclitaxel for 24 h. Protein extracts were blotted for total and phospho-H2AX (Ser^139^) to assess DNA Damage Response (DDR) and for total and phospho-p53 (Ser^15^) to assess p53 activation. Pan-Actin was a reference protein (n = 3 biological replicates). (**B**) RT-sqPCR analysis of total RNA extracted from RT4, RT112, T24, and TCCSUP cells, seeded at ~60% confluency and exposed to the indicated doses (0, 0.1, 1, 5, 10, and 50 μg/mL) of paclitaxel for 24 h. Expression of known p53 targets, *PUMA* and *NOXA* genes (mRNA levels), was analyzed to assess p53 activation. *GAPDH* mRNA expression was used as a control (n = 3 biological replicates). (**C**,**D**) Densitometry analysis of the obtained RT-sqPCR results. The density of *PUMA* and *NOXA* RT-sqPCR bands was analyzed and normalized to the density of *GAPDH* mRNA for each cell line and condition tested. A one-way ANOVA with Tukey’s test for multiple hypothesis correction was employed to examine the statistical significance of the results. Error bars represent means ± SD (n = 3 biological replicates) (n.s.: non-significant). See Supplementary Material for the original images of the Western Blots.

Microtubule dynamics disruption due to paclitaxel treatment impairs cell division and generates DNA damage and chromosomal instability, eventually leading to cell death [29,30]. Moreover, it is well known that the DNA Damage Response (DDR) pathway can further activate p53 and induce the expression of its transcriptional targets as a downstream effect [31]. To further elucidate the mechanism of the increased sensitivity of BLCA cells to microtubule dynamics perturbation, we treated our panel of BLCA cell lines with paclitaxel. We analyzed the DDR and p53 activation profiles using immunoblot for the phosphorylated forms of H2AX and p53 proteins, respectively. We uncovered a robust upregulation of phosphorylated H2AX (p-H2AX^Ser139^) even at the lower concentration(s) of paclitaxel used in all cell lines tested (Figure 6A). However, the total H2AX (reference/control) protein levels remained unaffected, especially at the lower drug doses (Figure 6A). 

Remarkably, in the high malignancy grade cell lines T24 (III) and TCCSUP (IV), we did not detect any phospho-specific, or total, p53 protein forms before and after paclitaxel administration, which confirms the lack of protein due to damaging mutations (Figure 6A). To our surprise, in the low malignancy grade cell lines RT4 (I) and RT112 (II), which have functional wild-type p53, there was no detectable upregulation of phospho-p53 (Ser^15^) or total protein levels observed upon paclitaxel treatment (Figure 6A). This indicates that p53 cannot be phosphorylated/activated downstream of DDR in this setting.

To further examine whether p53 is activated due to paclitaxel-induced DDR, we used RT-sqPCR as an orthogonal assay. We analyzed the mRNA expression/accumulation patterns of the p53 transcriptional targets *PUMA* and *NOXA*. Our analysis showed no transcriptional upregulation of the *PUMA* or *NOXA* genes, further confirming the absence of a p53 activation program following paclitaxel treatment of BLCA cells (Figure 6B–D).

Our results suggest that paclitaxel-induced microtubule dynamics perturbation in BLCA cells causes a robust DDR without engaging a p53 activation program.

### 3.6. Bladder Cancer Cell Sensitivity to Paclitaxel Is Independent of TP53 Activity but Highly Dependent on the Expression of DNA Repair Genes

**Figure 7:**
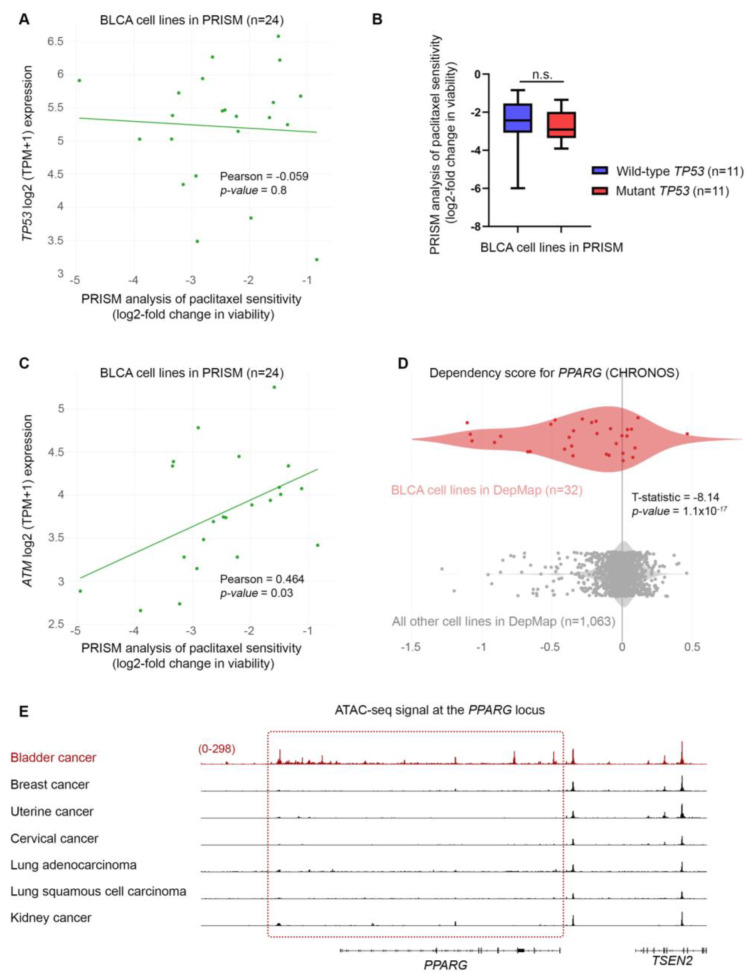
BLCA sensitivity to paclitaxel is independent of p53 activity but highly dependent on the expression of *ATM* and *PPARG*. (**A**) PRISM analysis for the sensitivity of BLCA cells to paclitaxel (log2 fold change in abundance of barcoded cells at d5 vs. d0) plotted against the expression of *TP53* mRNA (log2 TPM + 1). (**B**) BLCA cell lines in PRISM analysis were grouped based on their *TP53* (mutational or not) status, and the median sensitivity to paclitaxel was plotted. (**C**) PRISM analysis for the sensitivity of BLCA cells to paclitaxel (log2 fold change in abundance of barcoded cells at d5 vs. d0) plotted against the expression of *ATM* mRNA (log2 TPM + 1). (**D**) Two-class comparison of the CHRONOS score for *PPARG* between DepMap BLCA cell lines (n = 32) and all other cell lines in the database (n = 1063). (**E**) Normalized ATAC-seq. tracks of the *PPARG* gene locus in seven representative samples from various cancer types predicted to be dependent on *PPARG* and sensitive to paclitaxel (also, see Figure 2B and Figure 5A). Kidney cancer is used as a negative control. FC: Fold Change.

Our intriguing result of DDR without p53 activation in BLCA cells following paclitaxel treatment prompted us to revisit and further investigate the PRISM data. We first asked whether the sensitivity of BLCA cells to paclitaxel is correlated with the expression of *TP53*. We focused on the results of the primary PRISM screen and plotted the expression of *TP53* mRNA in relation to log2-fold changes for all BLCA cell lines included in the analysis (n = 24). We found no association between the expression of *TP53* and the sensitivity of the cells to paclitaxel (Figure 7A). Then, we investigated the status of the *TP53* gene in the BLCA cell lines used in the PRISM screen based on data from the Cancer Cell Line Encyclopedia (CCLE) [32]. We categorized the cell lines into two groups based on whether they have wild-type or mutant p53 (Table S4) and plotted the log2-fold survival changes for each group. Again, we found no correlation between the status of *TP53* and the response of BLCA cells to paclitaxel (Figure 7B), in line with our MTT and immunoblot results.

**Figure 8:**
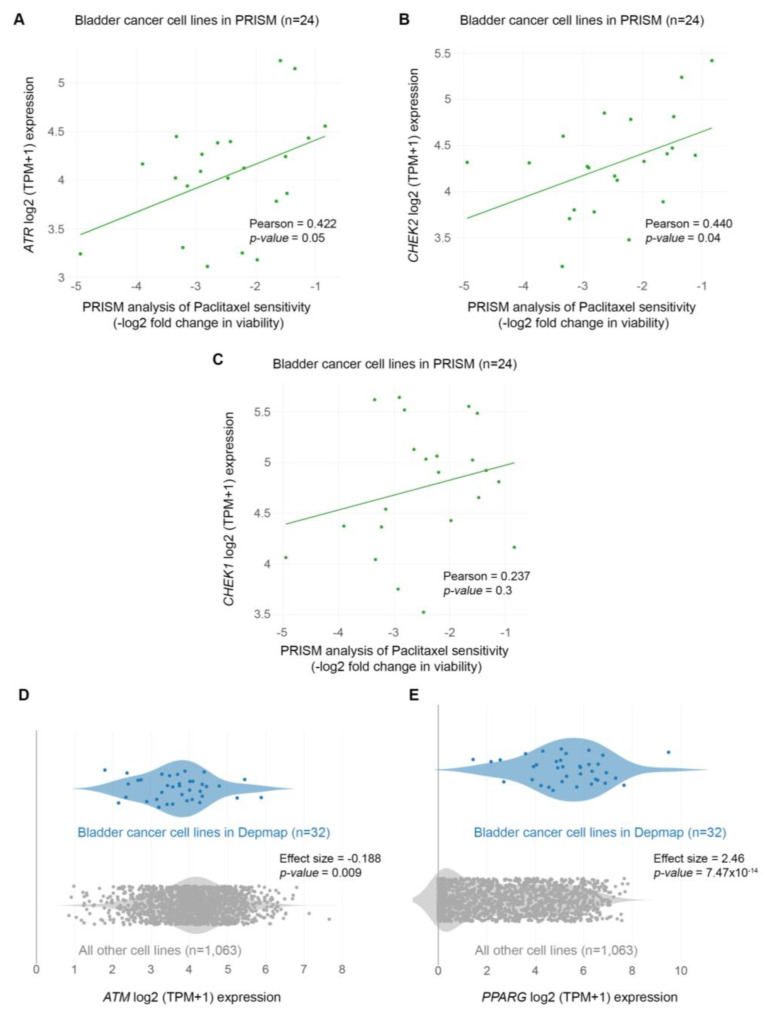
BLCA sensitivity to paclitaxel depends on the expression of DNA repair genes. (**A**) PRISM analysis of the sensitivity of BLCA cells to paclitaxel [Log2 (TPM + 1) = Log2-FC] plotted against the expression of *ATR* mRNA (RNA-seq.). (**B**) PRISM analysis of the sensitivity of BLCA cells to paclitaxel [Log2 (TPM + 1) = Log2-FC] plotted against the expression of *CHEK2* mRNA (RNA-seq.). (**C**) PRISM analysis of the sensitivity of BLCA cells to paclitaxel [Log2 (TPM + 1) = Log2-FC] plotted against the expression of *CHEK1* mRNA (RNA-seq.). (**D**) Two-class comparison of mRNA expression (RNA-seq.) for the *ATM* gene between DepMap BLCA cell lines (n = 32) and all other cell lines in the database (n = 1063). (**E**) Two-class comparison of mRNA expression (RNA-seq.) for the *PPARG* gene between DepMap BLCA cell lines (n = 32) and all other cell lines in the database (1063). FC: Fold Change.

Given the robust upregulation of phospho-H2AX following paclitaxel treatment (Figure 6A), we next asked whether there is an association between sensitivity to the drug and the expression of genes encoding DNA repair enzymes. Since microtubule dynamics perturbation caused by paclitaxel induces mainly DNA double-strand breaks (DSBs), we focused on the expression of ATM, ATR, CHEK1, and CHEK2 kinases (Figure 7C and Figure 8A–D). Our analysis showed lower log2-fold changes, indicating higher sensitivity to paclitaxel, correlated with lower expression of *ATM*, *ATR*, *CHEK1,* and *CHEK2* genes (mRNA levels). Specifically, we found a strong and significant association between the expression of ATM and CHEK2 kinases and the sensitivity of BLCA cell lines (n = 24) to paclitaxel (Figure 7C and Figure 8B). This further highlights the importance of the DSB repair pathway in the sensitivity of BLCA to paclitaxel.

Since the DepMap analysis revealed that BLCA cell lines were the most sensitive to paclitaxel, we hypothesized that the expression of DDR kinases would be overall lower in these cell lines compared to all other cell lines in DepMap. Indeed, when analyzing the expression of DDR kinases using the DepMap data, we found that BLCA cell lines have significantly lower expression of the *ATM* gene than all other cell lines in DepMap (Figure 8D).

Altogether, our results indicate that BLCA cells depend on DNA repair enzymes to ameliorate the toxic effects of paclitaxel-induced DNA damage, independently of the downstream activation of the p53 “genotoxic sensor”.

### 3.7. Bladder Cancer Cells Are Highly Dependent on PPARG Expression for Survival and Growth

Based on the association between sensitivity to paclitaxel and expression of DNA repair kinases, we asked if any of these enzymes scored as dependencies in BLCA cell lines. Interestingly, although none of the DNA repair enzymes were among the significant hits in DepMap, we found that *PPARG* (Peroxisome Proliferator-Activated Receptor γ) scored as the top dependency (Figure 7D and Table S2). PPARG has been shown to play a role in DNA repair, DNA damage-mediated activation of Caspase-8, and apoptosis [33]. Since BLCA cell lines are among the most sensitive to paclitaxel, we asked if there is a differential expression of the *PPARG* gene in BLCA cells. We found that BLCA cell lines express significantly higher *PPARG* levels than all other cell lines tested in DepMap (Figure 8E).

Finally, to investigate if the selective dependency of BLCA cells to *PPARG* and its higher expression in this lineage are driven by an open chromatin state, we investigated the normalized ATAC-seq. signal of BLCA primary cancer cells compared to other cancers at the *PPARG* locus. Surprisingly, when comparing BLCA to breast, uterine, cervical, and lung cancer cells, known to be highly sensitive to paclitaxel, we uncovered a selectively open chromatin state at the *PPARG* locus of BLCA cells compared to the other cancers tested (Figure 7E; kidney cancer was used as a negative control). Remarkably, an open chromatin state is detected in the coding and upstream/regulatory regions of the *PPARG* locus (Figure 7E).

Collectively, our results strongly suggest that the dependency of BLCA cells on PPARG underlies its role in DNA damage-mediated apoptosis. In contrast, the high sensitivity of these cells to paclitaxel is, at least partially, dependent on the high expression of PPARG.

## 4. Discussion

The predictive value of Cancer Dependency Map (DepMap) CRISPR screens has been validated in numerous publications [34]. At the same time, recently, Chetverina et al. (2023) demonstrated the power of DepMap data in uncovering novel “supertargets” for cancer therapy [35]. In this study, we comprehensively analyzed data generated by the DepMap and PRISM projects to uncover novel genetic dependencies and sensitivities to compounds, specifically for BLCA. To our surprise, genetic and chemical screens converged on the importance of microtubule dynamics for BLCA cells’ survival and the robust anticancer effects of perturbing these dynamics, specifically for BLCA.

We first focused on the genetic dependencies specific to BLCA cells and the biological processes they control. Many of the genes scored as dependencies are involved in microtubule dynamics, revealing the importance of this biological process for the survival of BLCA cells. Microtubules are dynamic structures that play crucial roles in cell homeostasis and, most importantly, cell division, as they form the mitotic spindle that facilitates chromosomal segregation during mitosis and meiosis [36,37]. During cell division, microtubules are formed by the polymerization of alpha and beta tubulin subunits, and at the end of metaphase, they depolymerize by the reverse reaction. Numerous studies have shown the importance of microtubule dynamics for proper cell division and that perturbing the proper and timely formation and depolymerization of the mitotic spindle results in errors in cell division and, ultimately, cell death [37]. Given the dependence of BLCA cells on microtubule dynamics, we next asked whether these cells are sensitive to agents that disrupt this dynamic. We focused on paclitaxel (distributed and sold under the brand name Taxol), the most well-characterized and widely used agent that perturbs microtubule dynamics.

Paclitaxel binds to beta-tubulin and blocks microtubule depolymerization. This stabilizes microtubules and prevents the proper execution of anaphase and telophase, leading to errors in chromosomal segregation and, ultimately, cell death [38]. Thus, we investigated data generated by the PRISM repurposing project. We asked what the sensitivity of BLCA cells to paclitaxel is and how it is compared to other cancer types, especially breast, ovarian, and lung, treated with paclitaxel as a standard of care. In perfect alignment with the CRISPR screen/dependency data, we found that BLCA cells are among the most sensitive to paclitaxel and significantly more sensitive than breast, ovarian, and lung cancer cells. Moreover, we further expanded our analysis with the in vitro treatment of four BLCA cell lines with a dose range of paclitaxel, which resulted in robust anti-proliferative responses.

Paclitaxel treatment can cause DNA damage and activate repair mechanisms to fix the damage and maintain genomic integrity. However, if the DDR is impaired or the DNA damage is too severe, cells can undergo apoptosis. Given the extremely high sensitivity of BLCA cells to paclitaxel, we tested markers of DDR in our system. We found a robust upregulation of the DNA DSB marker phospho-H2AX, but to our surprise, we did not observe an activation of p53, at least in the two wild-type cell lines for the *TP53* gene. Since we focused our analysis on the early stages of cell response to paclitaxel (24 h), it is possible that p53 is activated at a later time point and its status affects the later stage(s) of the response. However, since we detected activation of apoptosis, as determined by cleaved Caspase-8, -9, and -3, without transcriptional upregulation of the p53 targets *PUMA* and *NOXA* genes, it is evident that paclitaxel can induce apoptosis in BLCA cells without activating the p53 pathway. Moreover, when looking at the response to paclitaxel in the large collection of BLCA cell lines used in the PRISM project, we found no association between the response and the status of the *TP53* gene. This further confirms the p53-independent nature of the response.

It is well established that functional p53 can effectively mediate cell death, whereas mutant p53 can help cancer cells evade cell death and, thus, provide resistance to various cytotoxic agents. Although p53 is activated downstream of DDR, there are cases where a p53-independent pathway can mediate DNA damage-induced apoptosis by activating ATM and its downstream effector kinase, CHEK2 [39,40]. Given the lack of p53 activation, we asked if the response to paclitaxel in BLCA is associated with the expression of the DDR kinases ATM, ATR, CHEK1, and CHEK2, utilizing the transcriptomics data from the DepMap project. Our analysis clearly showed that the response to paclitaxel is strongly and significantly correlated with the expression of ATM and CHEK2. Moreover, BLCA cells have a significantly lower expression of ATM than all other cancer cell lines, potentially contributing to their enhanced sensitivity to paclitaxel. Interestingly, paclitaxel has been shown to bind BCL2 and block its anti-apoptotic action [24,25,27]. Most importantly, an open chromatin state has been observed for BLCA at the *BCL2* locus (Figure 5A). At the same time, BCL2 gene expression was downregulated in most BLCA cell lines treated with paclitaxel (Figure 5B,C). Therefore, it is possible that paclitaxel treatment in BLCA cells induces apoptosis by triggering a DDR-mediated activation of caspases and inhibiting BCL2, likely both at the transcriptional and post-translational levels (Figure S4).

The DepMap analysis revealed that the top dependency in BLCA is PPARG, a transcription factor that plays diverse roles in regulating cellular responses to DNA damage and apoptosis [41]. Paclitaxel treatment induces DNA damage, leading to the activation of the DDR pathway. This activation can upregulate the expression of PPARG, which can critically contribute to mediating apoptosis in response to DNA damage. Furthermore, PPARG is involved in regulating paclitaxel-induced apoptosis through its ability to regulate the expression of genes controlling apoptosis, oxidative stress, mitochondrial dysfunction, and cell cycle [42,43,44,45,46]. Therefore, it is possible that the strong dependence of BLCA on PPARG underlies its crucial role in maintaining genomic integrity and promoting cell survival. Due to their low expression of ATM and high expression of PPARG, BLCA cells are more likely to undergo apoptosis than repair DNA damage when treated with paclitaxel.

Most importantly, our analysis of publicly available ATAC-seq. data from a variety of human cancers [15] revealed that several genetic dependencies are correlated with an open chromatin state for the associated genes (*KATNB1*, *BCL2*, and *PPARG*) (Figure 2B, Figure 5A and Figure 7E). This suggests that BLCA cells carry unique genetic and epigenetic dependencies that can be exploited for the mechanistic understanding and therapeutic targeting of the disease.

The strong correlation between sensitivity to paclitaxel and expression of DNA DSB repair enzymes, such as the ATM/ATR and CHEK1/2 kinases, underscores the need to further investigate the combination of paclitaxel with ATM/ATR inhibitors as novel therapeutic regimens in BLCA. Similarly, the combined administration of paclitaxel with PPARG inhibitors may also benefit BLCA clinical treatment. Most importantly, our major finding that the growth inhibitory effect of paclitaxel on BLCA cells is independent of p53 activity strongly suggests that paclitaxel can treat a wide range of BLCA types independently of their p53 genetic status.

## 5. Conclusions

Our study combined a variety of in silico and in vitro approaches in a collection of human BLCA cell lines representing different stages of the disease. It demonstrated the importance of microtubule dynamics for BLCA cell survival and growth. It also unveiled the therapeutic potential of disrupting the pathway’s integrity and functionality independently of p53 oncogenic activity (Figure S4).

## Data Availability

All data are contained within the article or Supplementary Materials.

## Supplementary Materials

The following supporting information can be downloaded at: https://www.mdpi.com/article/10.3390/cancers15143730/s1, Figure S1: BLCA cell lines are among the most sensitive to paclitaxel treatment; Figure S2: Paclitaxel treatment induces cell cycle arrest in BLCA cell lines; Figure S3: BLCA cell lines express high levels of pro-apoptotic genes; Figure S4: Mechanistic model; Table S1: DNA sequence and annealing/cycling parameters of PCR primers for RT-sqPCR; Table S2: Dependencies enriched in BLCA cell lines (n = 32) against all other cell lines in the DepMap database (n = 1063); Table S3: Gene ontology analysis of the 35 genes scored as dependencies in BLCA; Table S4: Mutational status of the *TP53* gene in the 24 BLCA cell lines included in the PRISM analysis. File S1: The original images of the Western Blots.
