# Supplementary material for: Microtubule Dynamics Deregulation Induces Apoptosis in Human Urothelial Bladder Cancer Cells via a p53-Independent Pathway
Source: Cancers (Basel). 2023 Jul 22;15(14):3730. doi: 10.3390/cancers15143730 (PMC10378115; doi:10.3390/cancers15143730)
Supplement: Supplementary file 1 [file cancers-15-03730-s001.zip › Drosos et al Supplementary Images.pdf]

cancers

MDPI

Article - Supplementary Images

Microtubule Dynamics Deregulation Induces Apoptosis in Human Urothelial Bladder Cancer Cells via a p53-independent Pathway

Yiannis Drosos[1*], Eumorphia G. Konstantakou[2], Aggeliki-Stefania Bassogianni[1], Konstantinos-Stylianos Nikolakopoulos[1], Dimitra G. Koumoundourou[1], Sophia P. Markaki[1], Ourania E. Tsitsilonis[3], Gerassimos E. Voutsinas[4], Dimitrios Valakos[5], Ema Anastasiadou[5], Dimitris Thanos[5], Athanassios D. Velentzas[1] and Dimitrios J. Stravopodis[1*]

[1] Section of Cell Biology and Biophysics, Department of Biology, School of Science, National and Kapodistrian University of Athens (NKUA), 15701 Athens, Greece; sbasogianni@gmail.com (A-S.B.); ksnikolakop@biol.uoa.gr (K-S.N.); dimikoum@biol.uoa.gr (D.G.K.); smarkak@biol.uoa.gr (S.P.M.); tveletz@biol.uoa.gr (A.D.V.)
[2] Massachusetts General Hospital Cancer Center (MGHCC), Harvard Medical School, Charlestown, Boston, Massachusetts (MA) 02114, USA; ekonstantakou@mgh.harvard.edu
[3] Section of Animal and Human Physiology, Department of Biology, School of Science, National and Kapodistrian University of Athens (NKUA), 15701 Athens, Greece; rtsitsil@biol.uoa.gr
[4] Laboratory of Molecular Carcinogenesis and Rare Disease Genetics, Institute of Biosciences and Applications (IBA), National Center for Scientific Research (NCSR) "Demokritos", 15310 Athens, Greece; mvoutsin@bio.demokritos.gr
[5] Center of Basic Research, Biomedical Research Foundation of the Academy of Athens (BRFAA), 11527 Athens, Greece; dvalakos@bioacademy.gr (D.V.); anastasiadou@bioacademy.gr (E.A.); thanos@bioacademy.gr (D.T.)
* Correspondence: ydrosos@biol.uoa.gr (Y.D.); dstravop@biol.uoa.gr (D.J.S.); Tel.: +30-210-727-4105 (D.J.S.)

Supplementary Images

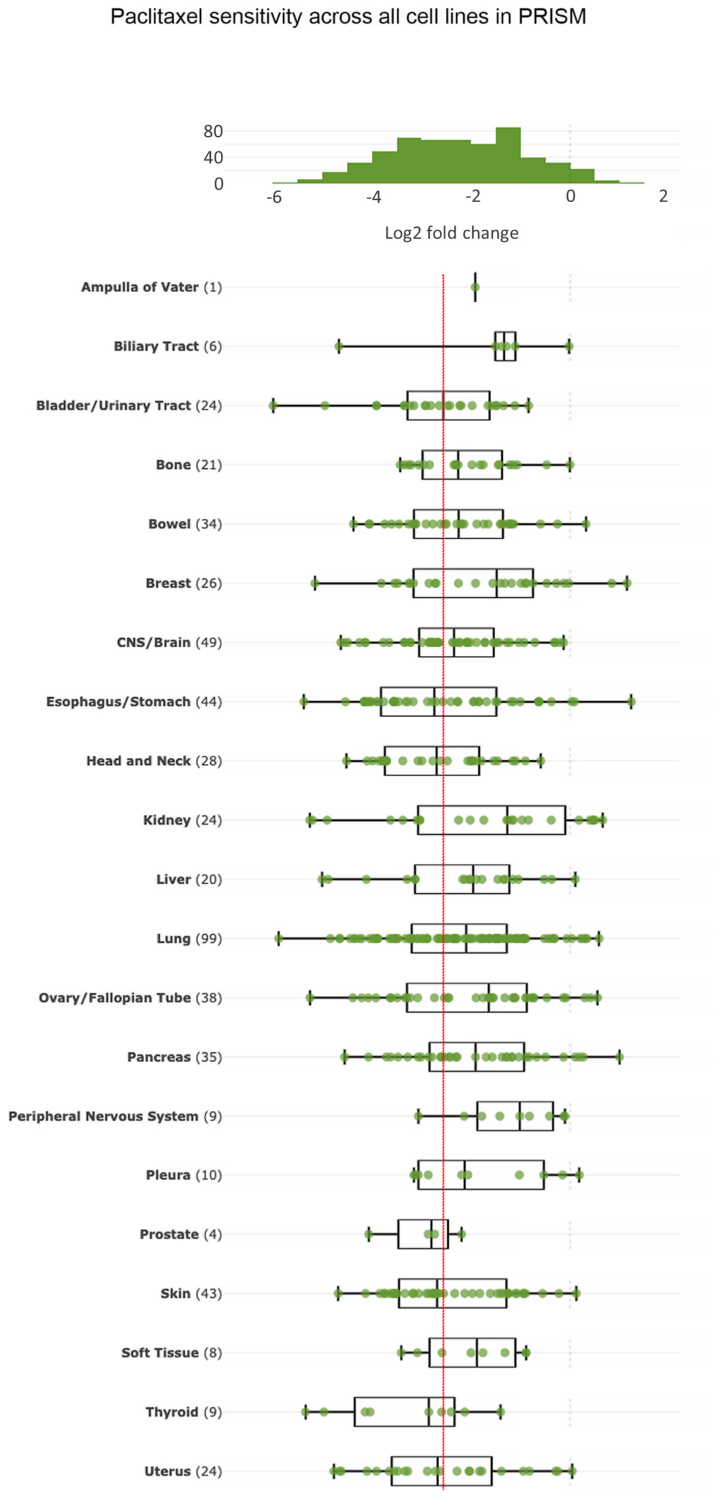

Figure S1. Cell lines sensitivity to paclitaxel treatment: BLCA cells are among the most sensitive ones. Viability analysis as measured by the log2 fold change of barcoded cells at d5 compared to d0 (see text for details) of paclitaxel treatment. In total, 499 cell lines from 21 lineages/cancer types were analyzed and the log2 fold changes for each lineage are plotted as box plots. The median log2 fold change in viability for BLCA cells (-2.55) was lower compared to lung (-2.1), breast (-1.56) or ovarian carcinoma (-1.65) where paclitaxel is a standard of care treatment.

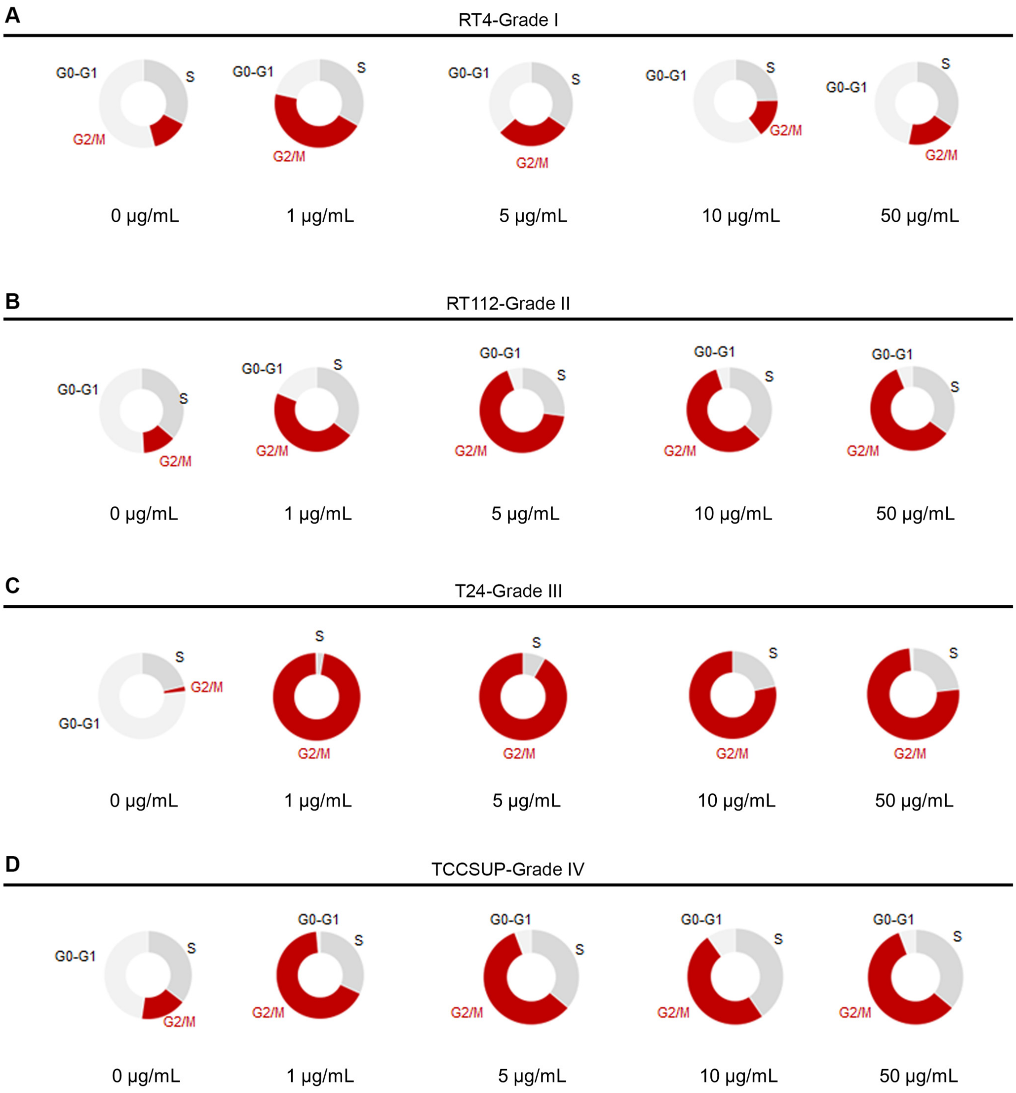

Figure S2. Paclitaxel treatment induces cell cycle arrest in BLCA cell lines. (**A**) Flow cytometry analysis of RT4 cells either untreated (0 µg/mL) or treated with a dose range of paclitaxel (1-50 µg/mL). (**B**) Flow cytometry analysis of RT112 cells either untreated (0 µg/mL) or treated with a dose range of paclitaxel (1-50 µg/mL). (**C**) Flow cytometry analysis of T24 cells either untreated (0 µg/mL) or treated with a dose range of paclitaxel (1-50 µg/mL). (**D**) Flow cytometry analysis of TCCSUP cells either untreated (0 µg/mL) or treated with a dose range of paclitaxel (1-50 µg/mL).

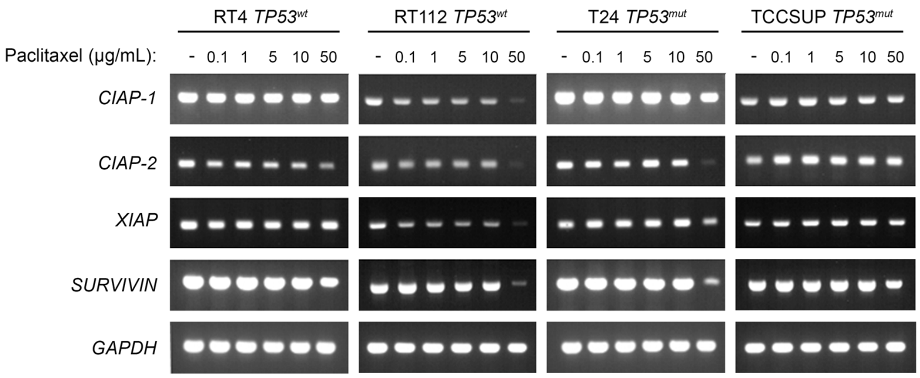

Figure S3. BLCA cell lines express high levels of pro-apoptotic genes. RT-sqPCR analysis of total RNA extracted from RT4, RT112, T24 and TCCSUP cells, seeded at ~60 % confluency and exposed to the indicated doses of paclitaxel for 24 h. Expression of *CIAP-1*, *CIAP-2*, *XIAP* and *SURVIVIN* indicate that BLCA cells have a fully functional pro-apoptotic machinery.

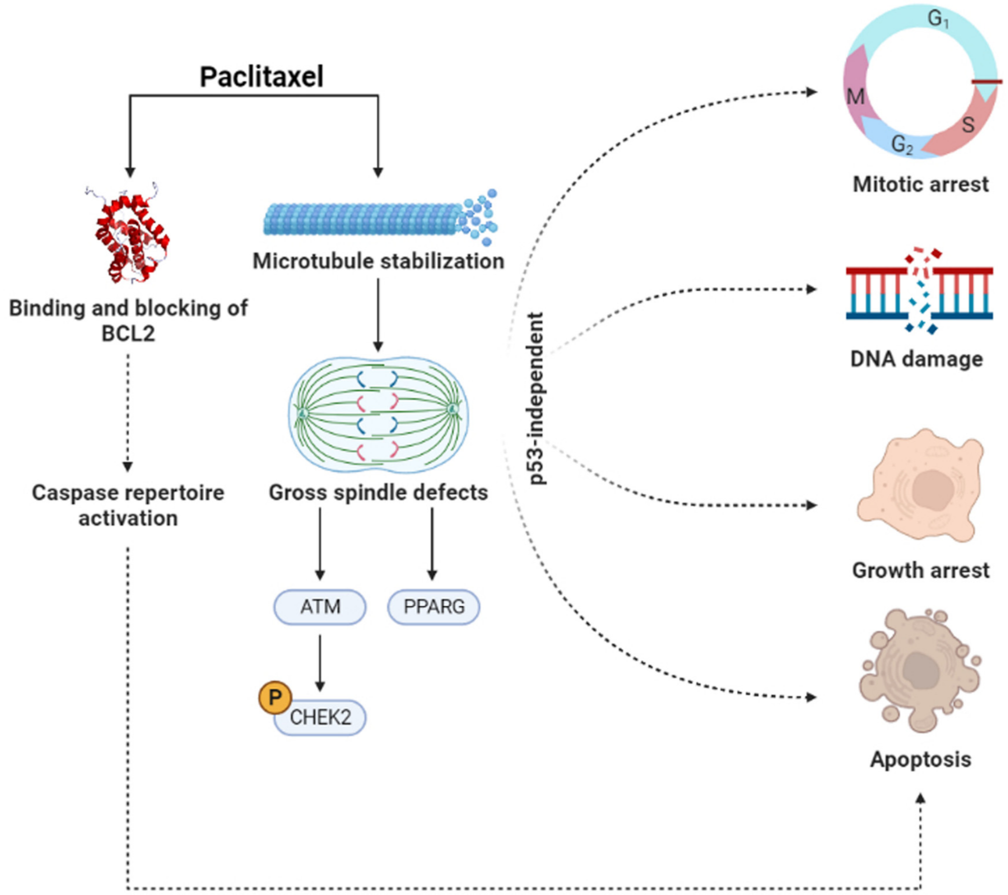

Figure S4. Mechanistic model. Paclitaxel treatment blocks microtubule depolymerization and induces an ATM-dependent but p53-independent DNA damage response, which results in G2-M cell cycle arrest, growth arrest and apoptosis. In parallel, paclitaxel can directly bind to BCL2 and block its anti-apoptotic activity, further inducing caspase-mediated apoptosis.
