# Supplementary figures and images for: Microtubule Dynamics Deregulation Induces Apoptosis in Human Urothelial Bladder Cancer Cells via a p53-Independent Pathway
Source: Cancers (Basel). 2023 Jul 22;15(14):3730. doi: 10.3390/cancers15143730 (PMC10378115; doi:10.3390/cancers15143730)

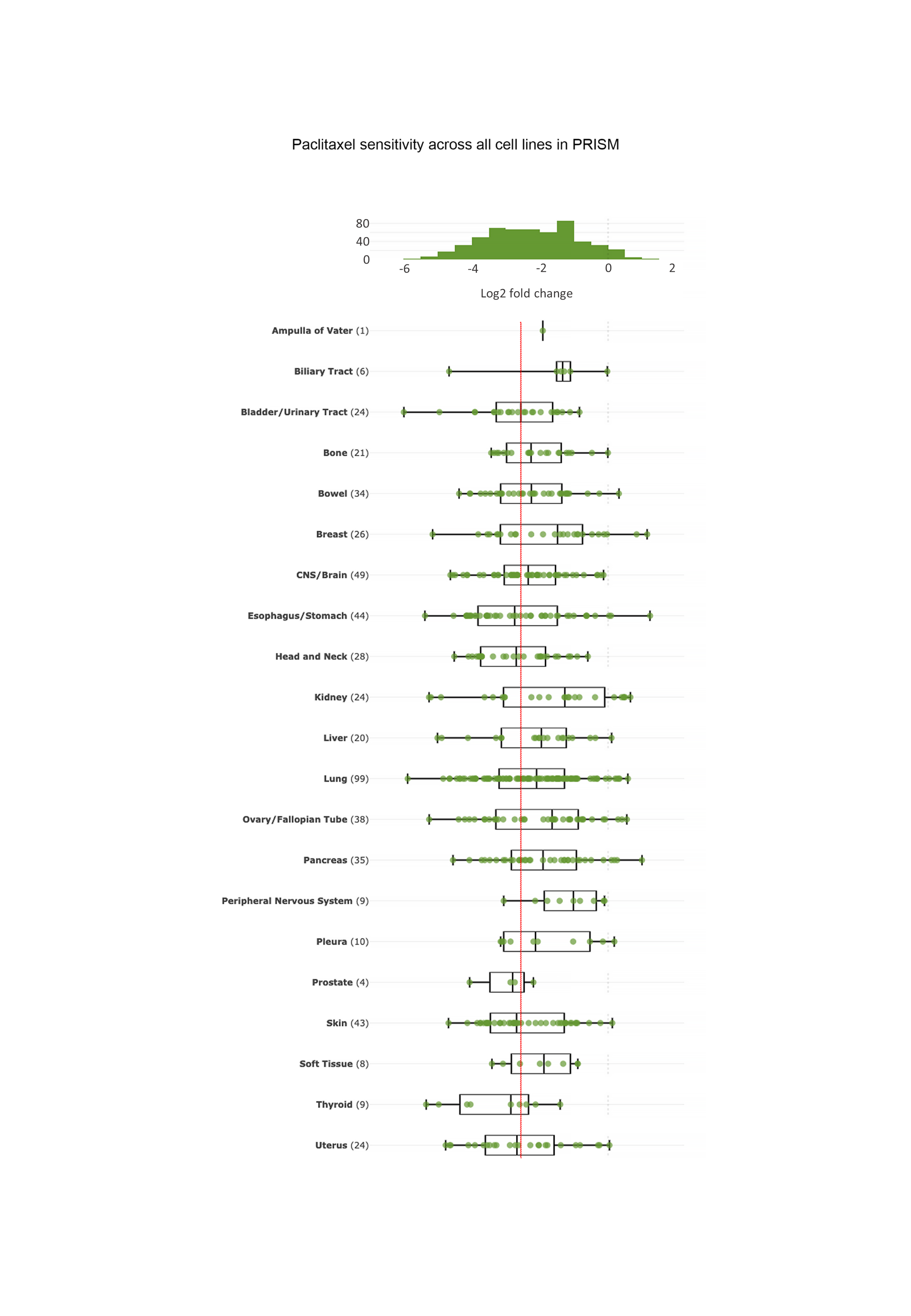

Supplement: Supplementary file 1 [file cancers-15-03730-s001.zip › Figure_S1.TIF]

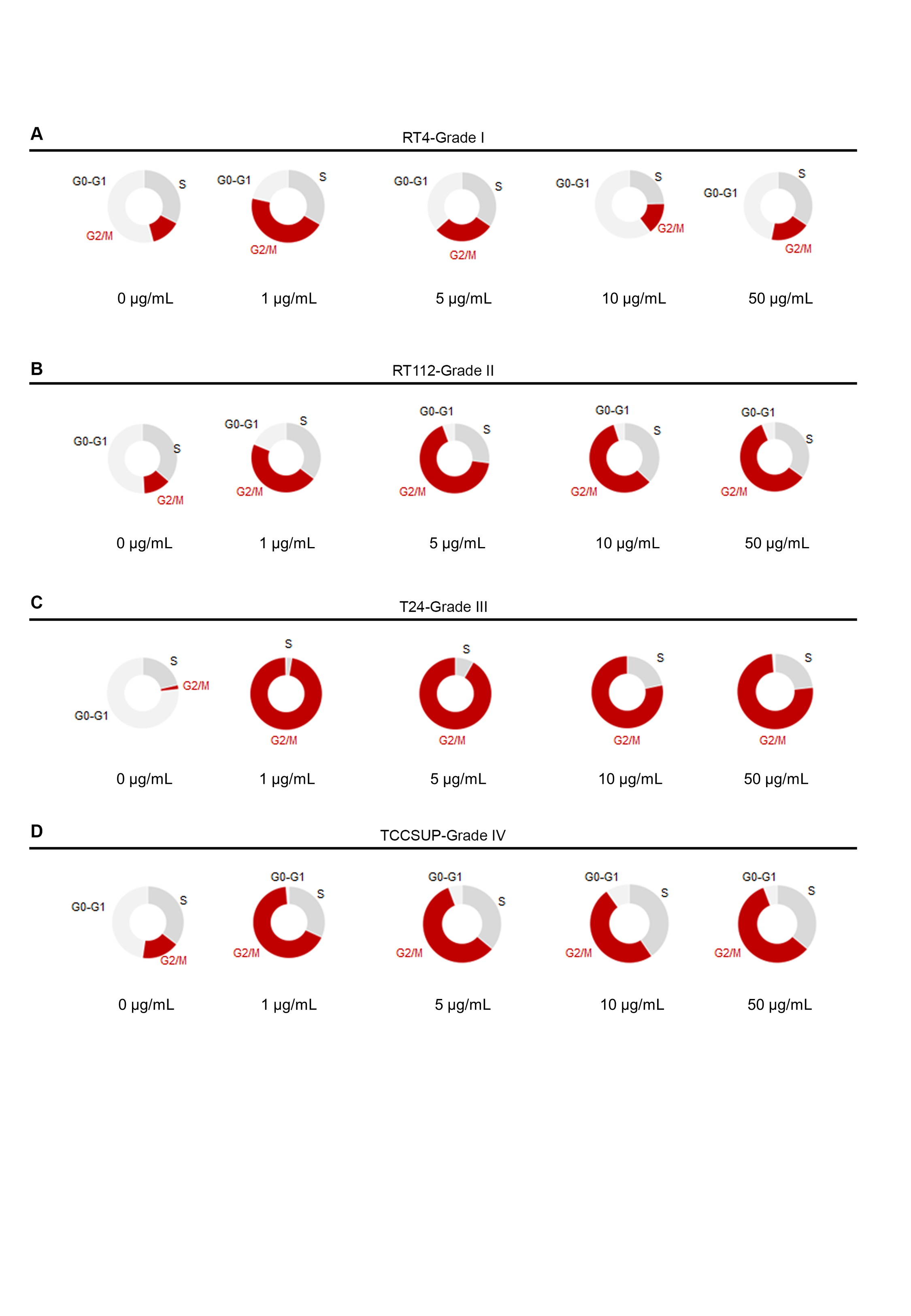

Supplement: Supplementary file 1 [file cancers-15-03730-s001.zip › Figure_S2.TIF]

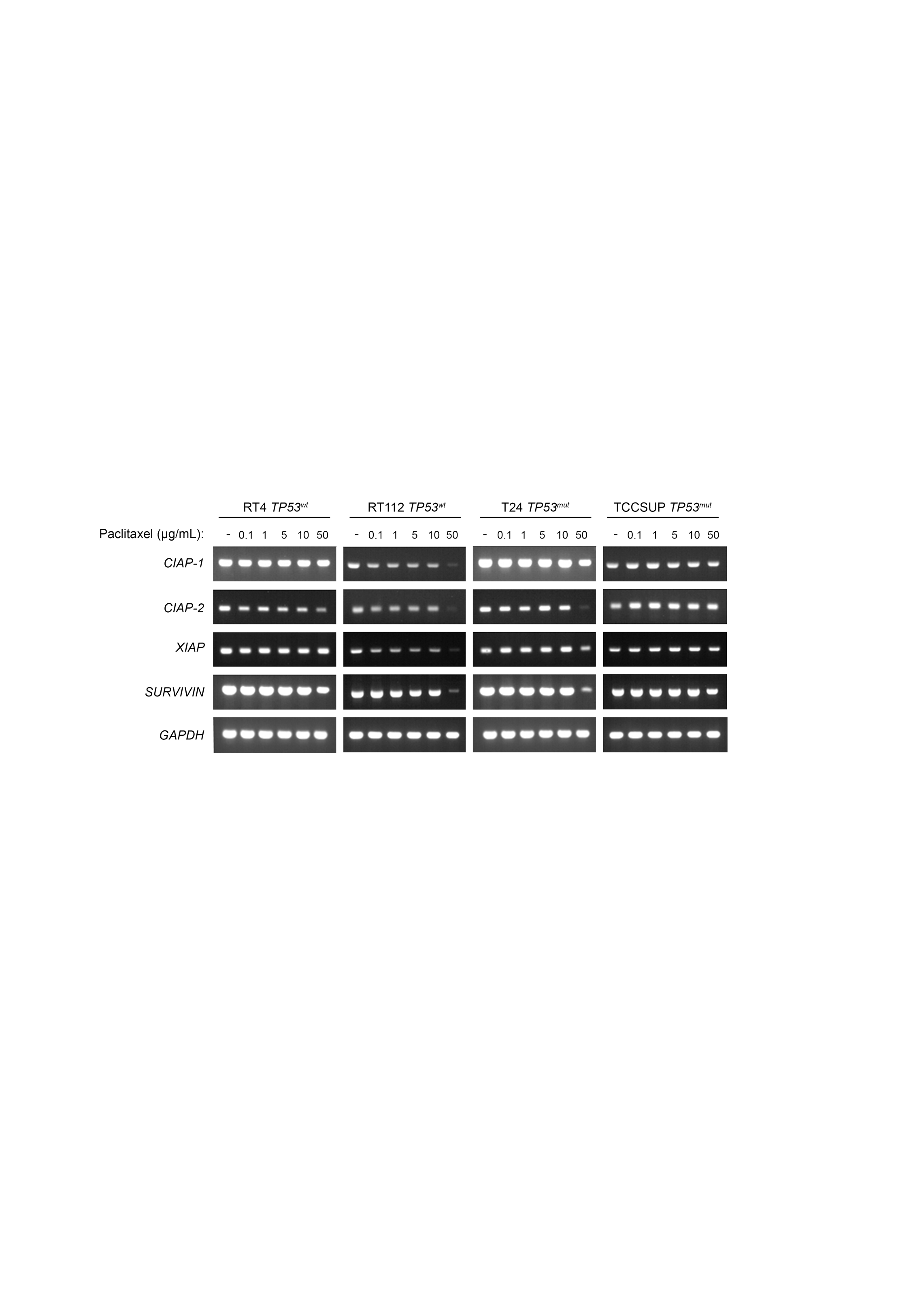

Supplement: Supplementary file 1 [file cancers-15-03730-s001.zip › Figure_S3.TIF]

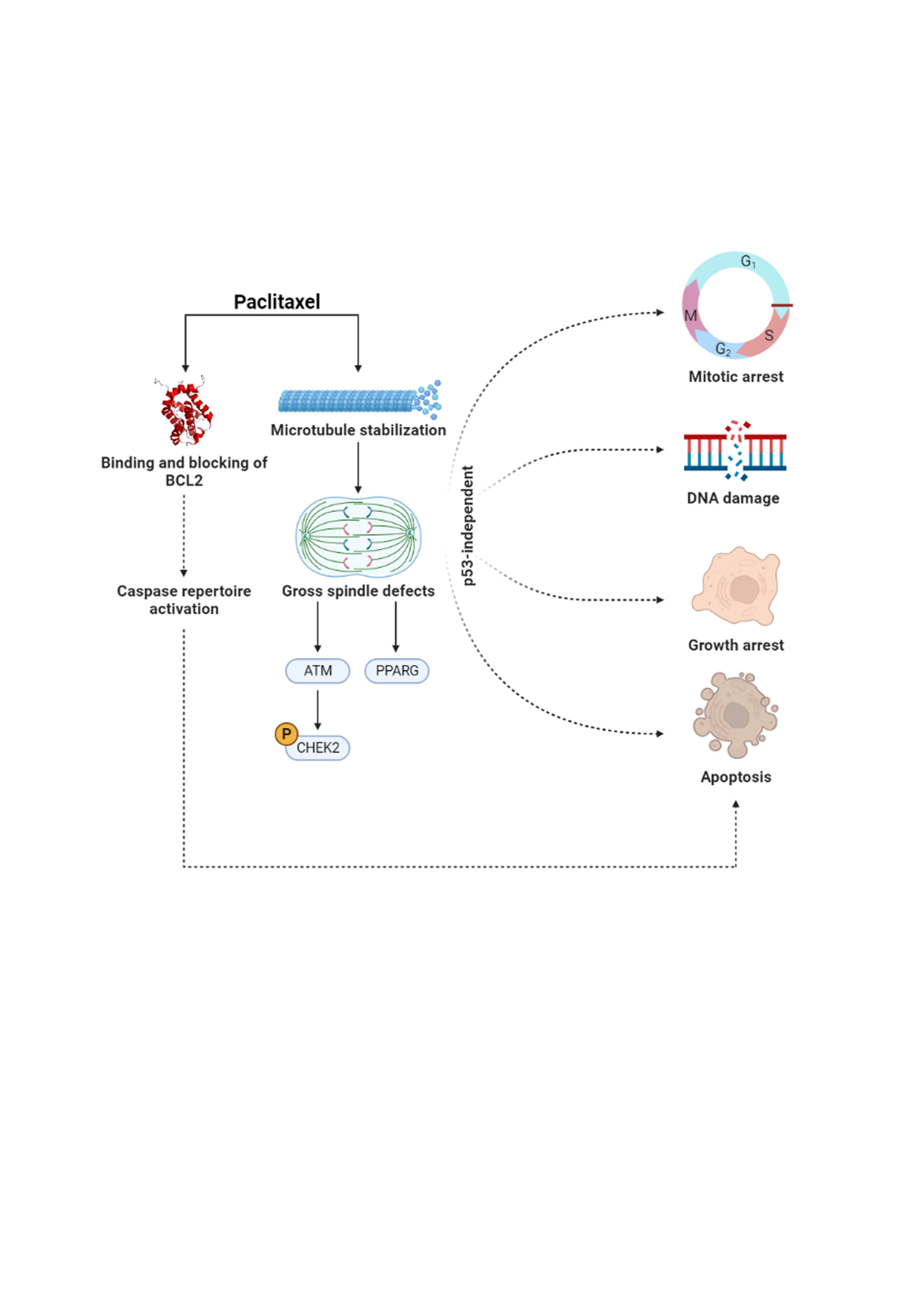

Supplement: Supplementary file 1 [file cancers-15-03730-s001.zip › Figure_S4.TIF]

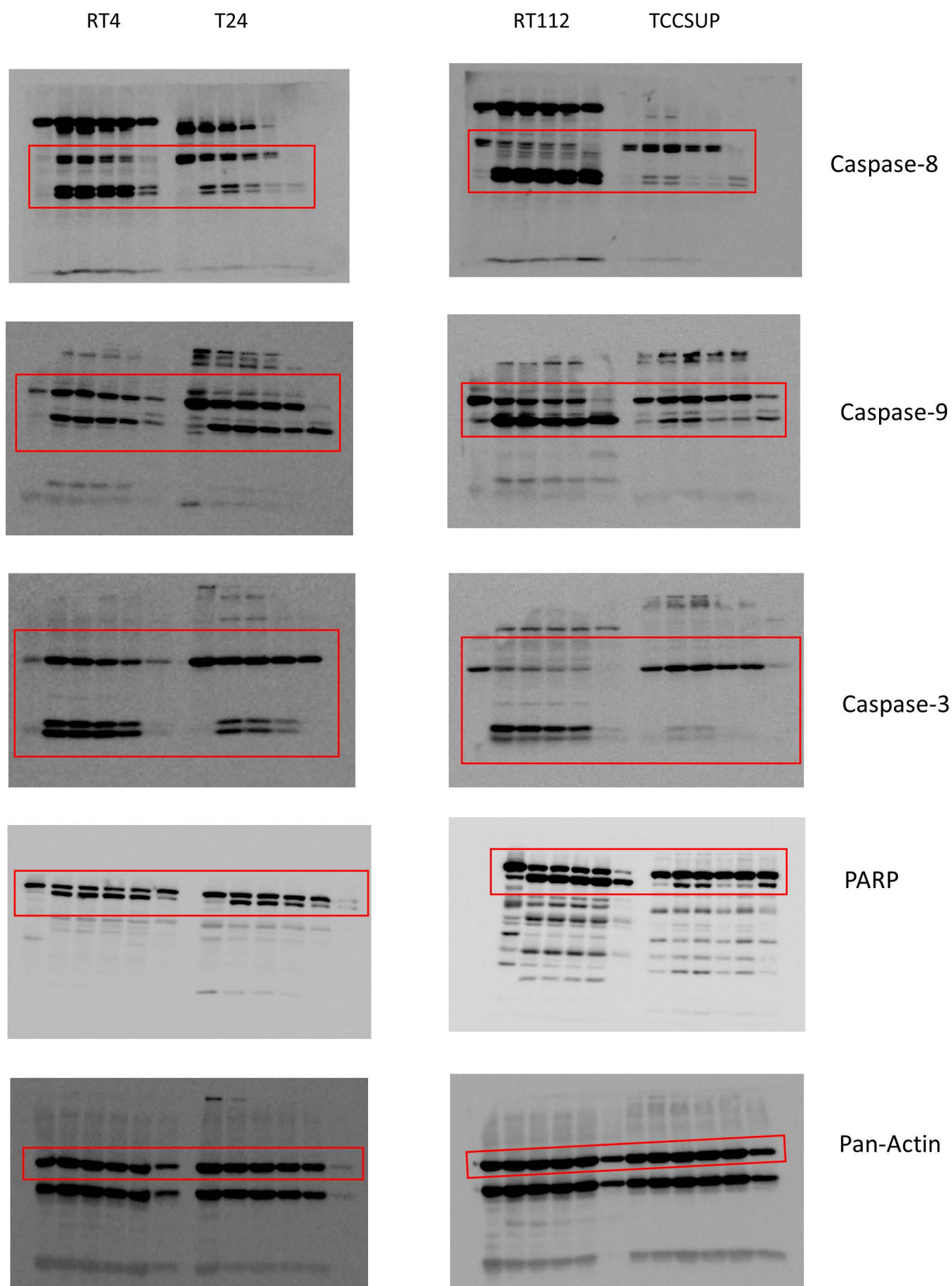

Figure 4A - original images of the Western Blots

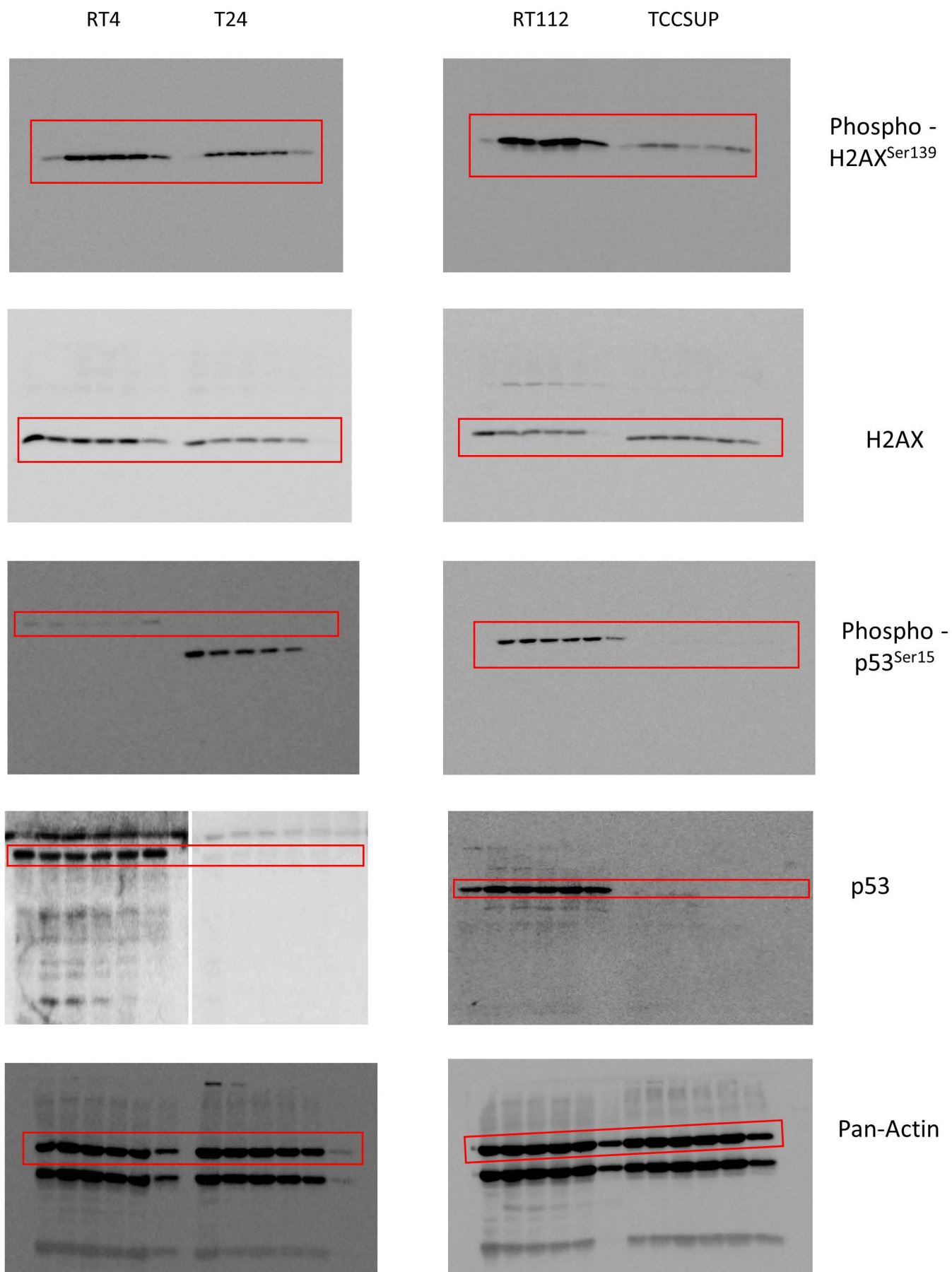

Figure 6A - original images of the Western Blots

Supplement: Supplementary file 1 [file cancers-15-03730-s001.zip › File S1.pdf]
